# Rehabilitation of torture survivors in five countries: common themes and challenges

**DOI:** 10.1186/1752-4458-4-16

**Published:** 2010-06-18

**Authors:** Helen McColl, Craig Higson-Smith, Sarah Gjerding, Mostafa H Omar, Basma Abdel Rahman, Mona Hamed, Aida S El Dawla, Miriam Fredericks, Nicole Paulsen, Gugu Shabalala, Carmen Low-Shang, Fernando Valadez Perez, Liliana S Colin, Aurora D Hernandez, Eliomara Lavaire, Arely PA Zuñiga, Lucia Calidonio, Carmen L Martinez, Yasser Abu Jamei, Zeyad Awad

**Affiliations:** 1 International Rehabilitation Council for Torture Victims, Borgergade 13, P.O. Box 9049, 1022 Copenhagen K., Denmark; 2 South African Institute for Traumatic Stress, 302 Ideal Village, 30 Hannaben Street, Johannesburg 2198, South Africa; 3 El Nadim Centre for the Management and Rehabilitation of Victims of Violence, 3A Soliman El Halabi Street from Rasmis Street, Cairo, Egypt; 4 Trauma Centre of Survivors of Violence and Torture, Cowley House, 126 Chapel Street, Woodstock 7925, Cape Town, South Africa; 5 Collective Against Torture and Impunity, Pitágoras 1210, 16 Col. Del Valle, 03100 Mexico DF, Mexico; 6 Centre for Prevention, Treatment and Rehabilitation of Victims of Torture and their Relatives, Col. La Reforma, Calle Principal 109, Contiguo a Centro IDEAL, Tegucigalpa M.D.C., Honduras; 7 Gaza Community Mental Health Programme, Sheikh Ejleen-El Rasheed Street, P.O. Box 1049, Gaza City, Gaza Strip, Palestinian Occupied Territories

## Abstract

**Background:**

Torture continues to be a global problem and there is a need for prevention and rehabilitation efforts. There is little available data on torture survivors from studies designed and conducted by health professionals in low income countries. This study is a collaboration between five centres from Gaza, Egypt, Mexico, Honduras and South Africa who provide health, social and legal services to torture survivors, advocate for the prevention of torture and are part of the network of the International Rehabilitation Council for Torture Victims (IRCT).

**Methods:**

Socio-demographic, clinical and torture exposure data was collected on the torture survivors attending the five centres at presentation and then at three and six month follow-up periods. This sample of torture survivors is presented using a range of descriptive statistics. Change over time is demonstrated with repeated measures analysis of variance.

**Results:**

Of the 306 torture survivors, 23% were asylum seekers or refugees, 24% were socially isolated, 11% in prison. A high level of traumatic events was experienced. 64% had suffered head injury whilst tortured and 24% had ongoing torture injury problems. There was high prevalence of symptoms of anxiety, depression, post traumatic stress as well as medically unexplained somatic symptoms. The analysis demonstrates a modest drop in symptoms over the six months of the study.

**Conclusions:**

Data showed that the torture survivors seen in these five centres had high levels of exposure to torture events and high rates of clinical symptoms. In order to provide effective services to torture survivors, health professionals at torture rehabilitation centres in low income countries need to be supported to collect relevant data to document the needs of torture survivors and to evaluate the centres' interventions.

## Background

Despite a range of international conventions and prevention instruments dating back to the end of the Second World War, torture is still practiced by at least 81 governments around the world [1]. The definition of torture is contested and different organisations define the concept more or less broadly. The most commonly used definition is that contained within the United Nations Convention Against Torture and Other Cruel, Inhuman and Degrading Treatment or Punishment (UNCAT) of 1985, which defines torture as,

any act by which severe pain or suffering, whether physical or mental, is intentionally inflicted on a person for such purposes as obtaining from him or a third person information or a confession, punishing him for an act which he or a third person has committed, or intimidating or coercing him or a third person, or for any reason based on discrimination of any kind, when such pain or suffering is inflicted by or at the instigation of a public official or other person acting in an official capacity. It does not include pain or suffering arising only from, inherent in or incidental to lawful sanctions [2].

What is not in dispute is that torture includes both physical and psychological methods of causing pain, distress or harm. Common forms of physical torture include beatings, burning, suspensions and stress positions, suffocation and drowning and electrical shocks. Psychological torture is most commonly seen in the forms of threats of execution and torture, threats against family members, mock executions, extended solitary confinement, sensory deprivation or overload, sleep deprivation, humiliation, and the forced participation in torture of others. Of course, most forms of torture involve both physical and psychological components, of which rape and other forms of sexual violence are clear examples.

Given the absence of comprehensive torture treatment in most developing countries, some torture survivors turn to one of the many non-governmental torture rehabilitation centres around the world for assistance. These centres are typically founded and run by small groups of health professionals and human rights activists, often at great personal cost and risk. This paper reports on a collaborative study of torture rehabilitation undertaken by five such centres, namely:

1. *El Nadim Centre for Psychological Management and Rehabilitation of Victims of Violence *in Egypt;

2. *Gaza Community Mental Health Programme *(GCMHP) in the Palestinian Occupied Territories;

3. *Centro de Prevención, Tratamiento y Rehabilitación de las Víctimas de la Tortura y sus Familiares *(CPTRT) in Honduras;

4. *Colectivo Contra la Tortura y la Impunidad A.C. *(CCTI) in Mexico;

5. *Trauma Centre for Survivors of Violence and Torture *(TCSVT) in South Africa.

These centres are all members of the *International Rehabilitation Council for Torture Victims *(IRCT), a global network of 144 torture rehabilitation centres. These centres represent a diversity of socio-political and cultural contexts and their varied resources mean that a standardized rehabilitation program is neither possible nor desirable. Services may include the following:

• Medical (for both physical and mental health needs)

• Physiotherapy

• Counselling and psychotherapy: Individual, family and group work

• Legal, including medical legal reports for those seeking restitution for torture and assistance with the asylum seeking process

• Prison visits

• Outreach work to rural areas

• Practical help with basic needs (food, shelter, language lessons)

• Further social care and integration, including living skills, education and employment training

### The Socio-political context for torture survivors

Knowledge of the social and political context in which torture and recovery occur is key to understanding the needs of torture survivors. For example, torture survivors construct and reconstruct the meaning of their experiences and this is an important component of their recovery. How this meaning is constructed depends in part on the socio-political context in which the torture and recovery occur.

In Egypt, the UN Special Rapporteur on Torture has reported that "torture is systematically practiced by the security forces, in particular the state security intelligence. In spite of denials of the government, the allegations of torture submitted by reliable, non governmental organizations consistently indicate that reported cases of torture are seen to be habitual, widespread and deliberate in at least a considerable part of the country." [3]

Torture in Gaza is closely related to the political situation which includes Israeli occupation and siege as well as a violent power struggle between the political factions Fatah and Hamas. Torture of Palestinians thus happens both during arrest by Israeli police and military, in Israeli prisons, as well as at the hands of the Palestinian political factions, and in the Palestinian prisons.

In Honduras, torture has been carried out as a mechanism of domination and social control since the time of colonialism. In the 1980s a US backed National Security Doctrine characterized by the use of death squads and military police to abduct, torture and murder political opponents as well as human rights and peace activists, was established. Violence was and continues to be legitimized from the highest levels of economic and political power.

Torture in Mexico is part of the structure of social inequity and responds to a doctrine of National Security similar to the one in Honduras. It is used for purposes of investigation, punishment, social demobilization and repression of ethnic minorities under the pretext of serving the purpose of counter insurgency in the continuous war against drug trafficking and organized crime. Political opponents of the government, syndicate members and leaders as well as leaders of lands rights, indigenous rights and other social movements, who openly criticize government actions, are targeted and "evidence" of their participation in drugs or arms trade is often planted on them or in their homes.

During apartheid in South Africa, torture and arbitrary deprivation of liberty were widespread and institutionalized. South Africans were tortured for their political beliefs and their efforts to bring about a democratic society. Unfortunately, many attitudes, practices and habits from the apartheid time have survived. The debate on torture in South Africa today therefore focuses especially on places where people are deprived of their liberty: prisoners, detainees in police custody, asylum seekers and refugees, children in secure care facilities, and patients in psychiatric hospitals. South Africa is also currently home to several hundred thousand refugees and asylum seekers, many of whom have been tortured in their countries of origin.

### Research on torture survivors

While the political contexts in which torture takes place may be very different, there is some evidence to suggest that the consequences for individuals and families are consistent across different settings. A recent meta-analysis of surveys on conflict affected persons from 40 different countries found torture to be endemic in countries affected by pervasive conflict and that torture is associated with mental disorder across these settings [4]. The severe and lasting negative consequences to the physical and emotional wellbeing of torture survivors have been well documented across different political contexts [5,6] and [7]. Previous studies have often focused on specific populations, such as asylum seekers or post conflict persons, for example, Hyder et al [8] present work with Afghan refugees in Pakistan and Alexander [9] who writes about refugees from Burma living in very vulnerable circumstances in India and Malaysia. Some studies have shown elevated levels of depression, anxiety, Post Traumatic Stress Disorder (PTSD), somatic disorders as well as physical health needs [4,10].

A small number of studies of treatment outcome with certain groups of torture survivors in particular contexts have been published. These studies tend to focus on short term international led programmes, rather than on the contribution of the ongoing work of permanent local torture rehabilitation centres. Bolton et al [11] have demonstrated the effectiveness of Group Inter Personal Therapy for adolescent survivors of war and displacement in Northern Uganda. Weine et al [12] demonstrated the effectiveness of testimony therapy with refugees from Bosnia-Herzegovina to the United States of America. Carlsson's study in an IRCT centre in Denmark following up survivors of torture after nine months of multi-disciplinary service interventions showed no change in mental health outcomes [13]. A study looking at the effects of brief multi-disciplinary treatment in Nepal showed effectiveness in treating somatic symptoms and disability and increasing subjective functioning, but no clear improvement in mental health measures [14]. Similarly, group treatments focussing on empowerment and support have shown positive results [15] and [16]. However, precisely because of the difficulties of conducting research in under-resourced contexts and emergency situations, few methods have been systematically tested in different contexts [17].

There is a lack of mental health research and research infrastructure in low income countries. The Global Forum and WHO mapped mental health research in Low and Middle Income Countries (LMIC) [18]. Their study identified that persons exposed to violence and trauma were a prioritised population group for mental health research and recommendations were made for improving mental health research capacity in LMIC. This research is designed and implemented by clinicians working in low income countries in permanent long standing rehabilitation centres for torture survivors. They aimed to see if it was feasible, despite the challenges of heavy clinical loads and extremely difficult working environments, as seen above, to collect useful data on:

1. Demographics, torture exposure and clinical needs from the population of torture survivors attending their centres

2. The recovery trajectories of torture survivors receiving care in the centres, and any differences between these patients and those who drop out of treatment

## Methods

### Design and Statistical Analysis

Clinical Staff at the centres received basic training on research methods, and participated in the design and data collection in the course of their therapeutic work. They developed a standardized intake questionnaire, and then follow up questionnaires after three and six months. Data was collected on socio-demographics, trauma exposure and clinical needs of the torture survivors. Staff used a questionnaire previously developed for use with asylum seekers in the UK as a starting point to develop their own questionnaire [19]. Data collection took place from January to December 2008, both directly from patients and indirectly from patient records.

The selection of instruments by the five centres in the study was based on their acceptability, feasibility and language. They had to be brief, as staff and patient time was limited. The instruments used were the Beck Depression Inventory (BDI), the Harvard Trauma Questionnaire (HTQ) and the Camberwell Assessment of Needs, shortened version (CANSAS). The HTQ produces two scores of PTSD. HTQ (1-16) is a score derived from the first 16 items of the test which relate directly to the symptoms of PTSD as listed in the Diagnostic and Statistical Manual of Mental Disorders, 4^th ^Edition Revised (DSM-IVR). HTQ (1-30) is a total score which derives from the symptoms and further signs associated with the diagnosis of PTSD.

The demographic and assessment data was captured in the five centres using a standardized spreadsheet. Qualitative data from the Honduran and Mexican centres was translated from Spanish into English. The data from all five centres was then combined, cleaned and analyzed using the Statistical Package for the Social Sciences (SPSS - Version 10). Data queries and problems of consistency were referred back to the five centres for comment and correction. Frequency distributions and descriptive statistics were calculated for all variables and scales. Differences in exposure, functioning and symptomatology were calculated between key groups (for example: men and women, exiles [refugees and asylum seekers] and citizens) using chi-square tests and one-way analysis of variance. Differences between participants who left treatment (and so the study) before six months were also calculated on all baseline variables using chi-square and Mann-Whitney tests. Finally, changes in the number of symptoms, depression and PTSD scores over time were examined for those subjects who remained in the study using repeated measures analysis of variance. (Where the sphericity assumption was violated, the Huynh-Feldt correction was applied).

### The Sample

The sample comprised of 306 torture survivors presenting for individual treatment to the five rehabilitation centres during the period January to July 2008 (Table 1). These patients were followed up at three and six months following their initial presentation at the centres. Patients were excluded if they were under the age of 14 years or if the context prevented detailed individual documentation: those seen in remote settings which did not allow regular care and follow up, or where only group work was possible.

**Table 1:** Sample breakdown by country of treatment

Country	Number of Torture Survivors	%
Egypt	83	27
Honduras	79	26
Mexico	51	17
Palestine	33	11
South Africa	60	20
**Total**	**306**	**100**

Of the 306 torture survivors for whom baseline data was recorded, 148 (48%) dropped out before the three month assessment. A further 61 (20%) dropped out before the six month assessment. As a result treatment outcome analysis is based on the scores of 97 torture survivors who provided data at all three assessments (32%).

## Results

### Demographic Information

Of the total sample of 306 torture survivors 172 (56%) were male. The mean age was 37.7 years (SD 14.2). 70 (23%) were exiles (refugees, asylum seekers or illegal immigrants). Virtually all exiles came from African countries and were included in the samples from Egypt and South Africa. The most prevalent religions in this sample were Christianity (55%), and Islam (38%).

40% of these torture survivors described themselves as single, while 42% were married or in a permanent relationship. 10% described themselves as divorced or separated, and a further 8% were widowed. The mean number of children was 2.1, although 35% had no children, and a few individuals had up to nine children. 14% of the sample (predominantly exiles) reported that they did not know where members of their immediate family were.

When asked to rate their level of social support the majority (52%) described themselves as having daily support from family or friends. However, 24% were isolated, not having a member of their social network who they saw at least weekly. While the majority of torture survivors in this sample were living at home (82%), 11% were in prison, 5% were living in camps or shelters for exiles, and the last 2% were homeless.

The level of education of this sample of torture survivors is summarized in table 2.

**Table 2:** Education Level

Education level	Freq.	Percentage
Primary education not completed	63	(21%)
Primary education completed	81	(26%)
Secondary education completed	91	(30%)
Tertiary education qualification	32	(10%)
Unknown	39	(13%)
**Total**	**306**	**(100%)**

Whereas 65% of the sample described themselves as being entitled to work, only 45% of the total was employed. The others described themselves as retired (5%), taking care of the home and family (11%), studying (7%) or unemployed and searching for work (32%).

### Experiences of torture and other potentially traumatic experiences

These torture survivors reported having experienced an average of 5.4 (SD 4.1) of the different traumatic events listed in table 3 below. Approximately 10% of the sample had experienced more than 10 events. Men had higher levels of events than women (means of 5.8 events [SD 4.1] and 4.8 [SD 4.8]). Similarly exiles had higher numbers of different events than citizens (means of 6.9, [SD 5.0] and 4.9 [SD 3.7]). Table 3 summarizes the numbers reporting exposure to traumatic events. Where there are significant differences between the exposure of key groups (women, men, exiles, citizens), the group with higher exposure is noted.

**Table 3:** Prevalence of different types of traumatic exposur

Traumatic event	Number reporting event	% of total sample	Group with significant higher exposure
Torture	175	67	Men**
Being close to death	140	54	Men**
Forced separation from family	125	48	Women* & Exiles**
Serious injury	117	45	Men**
Ill-health without access to care	117	45	Men** & Exiles**
Imprisonment	110	42	Men** & Citizens*
Lack of food and water	99	38	Exiles **
Murder of family or friend	89	34	Exiles **
Unnatural death of family/friend	86	33	Exiles **
Lack of shelter	86	33	Exiles **
Forced isolation from others	82	32	Exiles **
Lost or kidnapped	63	24	Exiles **
Murder of stranger(s)	52	20	Exiles **
Combat	49	19	Men**
Rape or sexual abuse	46	18	Women** & Exiles**
Brainwashing	22	8	

p < 0.05 *, p < 0.01 **

While the exiles from Africa are significantly more at risk for a broad range of traumatic events, citizens are more likely to have suffered imprisonment. Men are more likely to have experienced torture, being close to death, serious injury, imprisonment and combat. Women are more likely to have experienced forced separation from family, and rape or sexual abuse.

### Head Injury

Data relating to head injuries sustained during torture was collected for 171 patients. Of these, 109 (64%) had sustained some form of head injury. 105 (61%) reported beatings to the head, 40 (23%) reported suffocation, and 18 (11%) reported drowning. 20 people (12%) reported losing consciousness during their ordeal.

### Service use

The rehabilitation centres participating in this study offer a range of different services, either directly or through referral to partner service providers. Table 4 summarizes the services used by torture survivors following an initial assessment during the study period.

**Table 4:** Service utilization

Service	Freq.	Percentage
Legal services	110	(42%)
Employment services	56	(21%)
Counselling or psychotherapy	51	(19%)
Social services	39	(15%)
Interpretation services	29	(10%)
Inpatient care	25	(10%)
Medical outpatient -- adult	24	(9%)
Crisis or emergency response	15	(6%)
Educational services	10	(4%)
Medical outpatient -- child and family	9	(3%)
Substance abuse services	3	(1%)
Alternative medical services	2	(1%)

### Physical health problems and injuries at initial presentation

Ongoing physical health concerns were noted in 61 cases (20%). These are broadly categorized in table 5.

**Table 5:** Ongoing General Health Concerns

Type of disorder	**Freq**.	Percentage
Circulatory disorders	24	(8%)
Endocrine disorders	12	(4%)
Neurological disorders	7	(2%)
Gastro-intestinal disorders	5	(2%)
Reproductive disorders	3	(1%)
Parasitic infections	2	(1%)
Orthopaedic problems	2	(1%)
Pulmonary disorders	2	(1%)

62 patients (20%) also presented with torture related injuries that continued to cause pain or distress. Table 6 summarizes the types of injuries reported.

**Table 6:** Ongoing Concerns relating to Injuries

Type of injury	Freq.	Percentage
Contusions	22	(7%)
Scarring	12	(4%)
Fractures	10	(3%)
Internal Injuries	6	(2%)
Mutilation	5	(2%)
Sprains, strains, dislocations	4	(1%)
Cut and stab wounds	3	(1%)
Gunshot wounds	3	(1%)
Burns	3	(1%)
Nerve damage	1	(.5%)

### Psychiatric assessment at initial presentation

Not all centres have doctors working at them, so some centres prefer not to make medical or psychiatric diagnoses unless absolutely necessary (and then some may do so in consultation with external physicians), for example for a medical-legal report. This in part reflects the professional make up of the centres and beliefs about the usefulness of a diagnosis. 196 clients (64%) did receive a diagnosis and these are detailed below (Table 7).

**Table 7:** Psychiatric Diagnosis

Diagnosis	Freq.	Percentage
Post Traumatic Stress Disorder	123	(40%)
Major Depressive Disorder	83	(27%)
Generalized Anxiety Disorder	28	(9%)
Adjustment Disorder	14	(5%)
Psychotic Disorder	5	(2%)
Somatoform Disorder	4	(1%)
Substance Abuse Disorder	4	(1%)
Obsessive Compulsive Disorder	2	(1%)
Conversion Disorder	2	(1%)
Delusional Disorder	2	(1%)
Borderline Personality Disorder	1	(.5%)
Panic Disorder	1	(.5%)
Dysthymia	1	(.5%)
Acute Stress Disorder	1	(.5%)
Mental Disorder due to General Medical Condition	1	(.5%)

A more detailed clinical picture is presented by the results of the clinical interview summarized in Table 8.

**Table 8:** Symptom Prevalence at Intake

Symptom Categories	Freq.	Percentage
*Anxiety related symptoms*		
General anxiety	206	(68%)
Panic attacks	40	(13%)
Social phobia	14	(5%)
Agoraphobia	8	(3%)
Obsessive/compulsive	4	(1%)
Simple phobia	3	(1%)

*Mood related symptoms*		
Depressed mood	190	(63%)
Irritability and anger	163	(54%)
Somatic symptoms	124	(41%)
Emotional Numbing	69	(23%)
Suicidal thoughts	34	(11%)
Elevated mood	29	(10%)
Passivity	12	(4%)

*Trauma related symptoms*		
Re-experiencing/intrusion	159	(52%)
Hyper-arousal	153	(50%)
Avoidance	137	(45%)
Dissociative symptoms	17	(6%)

*Symptoms relating to thought and perception*		
Poor concentration	90	(30%)
Loss of insight	28	(9%)
Dissociative symptoms	17	(6%)
Conceptual disorganization	14	(5%)
Disorientation	11	(4%)
Delusions	8	(3%)
Hallucinations	5	(2%)

Not unexpectedly, the symptom categories associated with anxiety disorders, post-traumatic stress disorder and mood disorders are the most prevalent. On average participating torture survivors reported symptoms in 5.9 of symptom categories listed in table 8 above (SD 2.8).

The HTQ and BDI provided similar results. PTSD scores from part 4 of the Harvard Trauma Questionnaire were collected from 71 torture survivors (from Mexico and Gaza) (Table 9).

**Table 9:** Harvard Trauma Questionnaire (HTQ) Scores

	mean	SD	Symptomatic for PTSD	Risk groups
HTQ (1-16)	2.32	0.8	30 (42%)	Women*
HTQ (1-30)	2.16	0.7	24 (34%)	Women*

p < 0.05 *, p < 0.01 **

Although these results suggest that more than a third of this sample is symptomatic for PTSD, they should be interpreted with caution. The cut off score is taken from international norms and a single test like the HTQ cannot be used in isolation to make diagnostic decisions. However, what these results do tell us is that a significant group of torture survivors in this sample report that they frequently experience many of the symptoms associated with PTSD.

Similarly high levels of depressive symptoms were measured using the Beck Depression Inventory. In order to ensure the best fit with available normal data the BDI I was used in Honduras and Mexico, and the BDI II was used in Egypt, Palestine and South Africa. The results are summarized in table 10.

**Table 10:** Beck Depression Inventory (BDI) Scores

	n	mean	SD	Moderate or Severe Symptoms	Risk groups
BDI I	122	14.2	9.8	29 (24%)	Women**
BDI II	113	27.8	15.2	73 (65%)	Women*

p < 0.05 *, p < 0.01 **

A particularly high prevalence of depressive symptoms was recorded in Palestine and Egypt. Again, these scores should not be taken as diagnoses of major depressive disorder but as an estimation of the high levels of depression related symptoms in this sample.

The total number of symptoms reported in the clinical interviews is significantly correlated with the total number of events experienced (r = 0.135, p = 0.024). Similarly scores on the HTQ and BDI tests are highly correlated (r = 0.840, p = 0.000) to each other. However, the HTQ and BDI scores are not significantly correlated with the number of events experienced.

### Recovery Trajectories

The results are for the torture survivors seen at both three and six months after initial assessment.

Torture survivors who left the study before the six month assessment were not different from those who completed all three measurements in terms of sex, marital status, number of dependents, level of education, employment status, baseline depression scores, number of unmet needs, or overall number of symptoms. However, those who stayed in treatment until the six month measurement tended to be younger (z = -2.26, p = 0.024) and to have fewer children (z = -2.28, p = 0.023). People who stayed in treatment long enough to complete all three measurements also reported a more traumatic experiences (z = -6.046, p = 0.000) and were more likely to report being raped ( χ^2 ^= 15.4, df = 1, p = 0.000) and/or being tortured ( χ^2 ^= 21.16, df = 1, p = 0.000). Refugees tended to stay in treatment longer ( χ^2 ^= 9.99, df = 1, p = 0.02) as did people who lived with fewer people (z = -2.09, p = 0.037), and who reported having less social support (z = -2.91, p = 0.004). Finally, people reporting more PTSD symptoms tended to leave treatment earlier (z = -4.514, p = 0.000).

The change in the total number of symptoms reported, the HTQ and BDI scores at initial assessment, three months and six months reveal in part the recovery trajectories of torture survivors.

**Figure 1:**
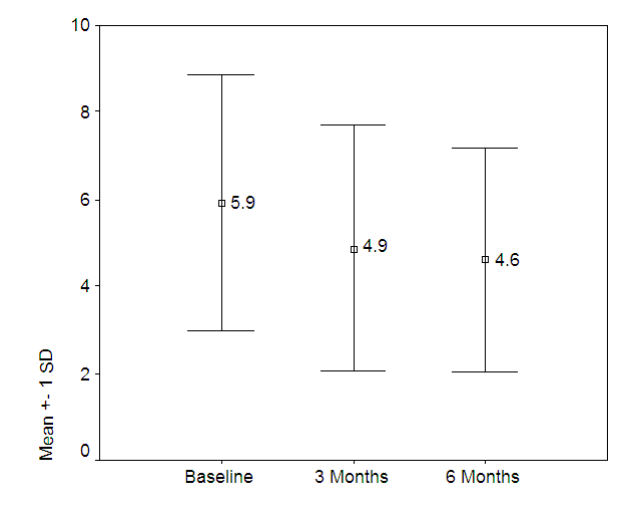
**Mean number of symptoms over time (n = 88)**.

**Figure 2:**
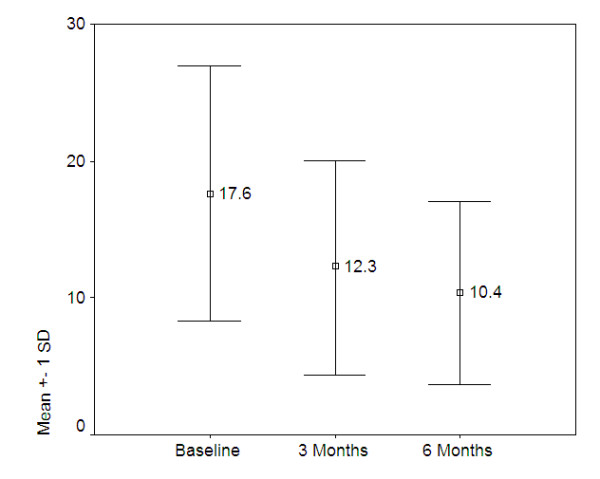
**Mean BDI-I scores over time, Honduras and Mexico (n = 32)**.

**Figure 3:**
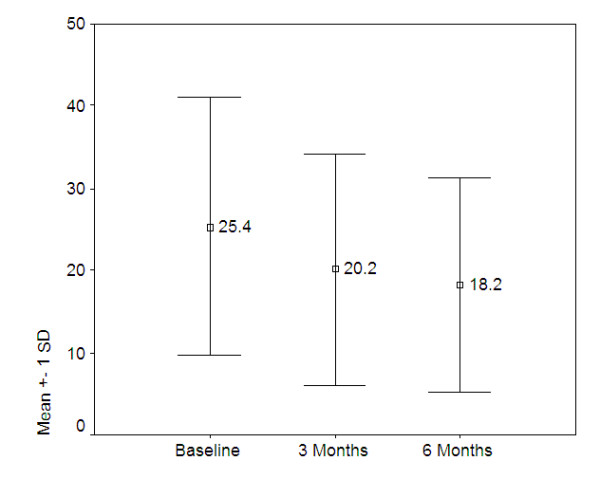
**Mean BDI-II scores over time, Egypt and South Africa (n = 47)**.

The mean number of symptoms dropped significantly over time (F[1.44, 126.78] = 25.86, p = 0.000) as illustrated in figure 1 below:

Measurements of depressive symptoms show similar significant drops for both the BDI-I (F[1.57, 48.78] = 13.349, p = 0.0000) and the BDI-II (F[1.17, 53.56] = 37.79, p-0.0000). These changes are presented in figures 2 and 3 below.

**Figure 4:**
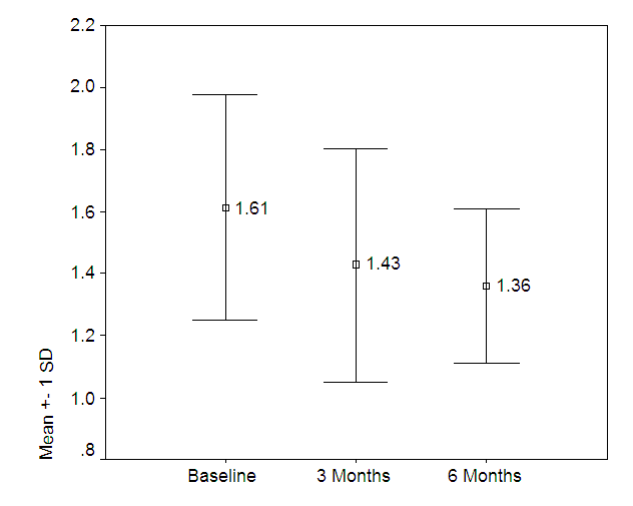
**Mean HTQ scores over time (n = 16)**.

Finally, although only the Mexican centre was able to collect HTQ data at all three measurement points, these results show significant improvement as illustrated in figure 4 below (F[2,30] = 5.097, p = 0.012).

## Discussion

Medical and psychological care of torture survivors very often takes place in extremely difficult social, political, and economic contexts, which has effect on both staff and patients. Commonly clinicians working with torture survivors are managing large case loads with limited access to space, equipment, medicines, and technical and emotional support. This situation is often exacerbated by humanitarian crises such as war, when conducting research is understandably low on the priority list. For example, four of the five centres in this study suffered from attacks on their staff and/or premises during the research period; the centre in Gaza suffered from a war with Israel and the Honduran centre from a military coup. Nevertheless, it is important that centres providing care to torture survivors use research to identify needs and evaluate the effectiveness of their work. Data can also play a crucial role in the prevention of torture through documenting allegations and evidence of torture and advocacy work.

The study was a complex undertaking and there are many limitations to the data which must be kept in mind when interpreting the findings. In relation to the sample it is important to remember that this is a clinical sample of torture survivors who have received individual assistance at one or other of the centres. The patients described in this study are in no way representative of torture survivors in general, the vast majority of whom do not access health services.

The high dropout rate must also be considered when interpreting the results. Unfortunately it is not possible to determine the range of reasons that resulted in the majority of patients not completing six months of care. However, these reasons are likely to include a lack of resources such as transport to reach the centres, and changes in life circumstances (such as moving to a different town, finding employment, or being released from prison). Of course it is also likely that some patients left treatment because they felt that they had made sufficient recovery, or because they felt that the treatment was not meeting their needs.

Differences in language, culture, training and the professional backgrounds of staff presented many challenges to the study. Across the various centres service provision and data capture were conducted in Arabic, Spanish and English. Furthermore, service providers came from different disciplines including medicine, psychology, nursing and social work. These disciplines all have their own assumptions and technical language, and even within disciplines training differs enormously between different countries. As such this study provided important lessons in international and interdisciplinary work. Although the researchers went to great lengths to ascertain standard meanings and interpretations in the data, it is unavoidable that in some cases the quality of data was compromised.

Furthermore, the cultural applicability and standardization of the various instruments used was questioned. For example questions about substance use and sexuality are potentially insulting in some cultural contexts and health professionals decided to exclude them in many patient interviews, thus leaving too little data to analyze. Where possible the most glaring of these differences was allowed for in the analysis but it must not be assumed that the results were not influenced by cultural differences. Similarly, normal data and cut off scores have not been adequately tested for the varied study populations.

Finally, although the assessments conducted were extensive there are some areas of functioning which were not adequately documented. A larger battery of tests was not feasible given the contexts in which service providers are working and so some compromises were required.

While the high levels of symptoms are not unusual in a clinical sample such as this one, they do underline the enormous and lasting distress experienced by many torture survivors. However, it is surprising to note that the HTQ and BDI scores are not significantly correlated with the number of events experienced, a reminder that trauma and depression related disorders are a result of each person's appraisal of life events, rather than of the events themselves.

The high prevalence of head injury (either due to beatings, drowning or suffocation) seen here is a particular challenge in the rehabilitation of torture survivors. Further research could investigate if head injury is adequately assessed and managed in torture survivors.

This study does provide an important picture of the demographic profile, living situations, experiences, and needs of torture survivors who seek assistance from treatment centres in these low income countries. This work also explores some of the difficulties of service provision (illustrated in part by the high dropout rate) and shows that where torture survivors are able to continue in care, modest but significant gains are likely, although whether due to treatment provided or external factors is not known. Finally, whilst carrying out this study we found enormous difficulties of collecting data on torture survivors where health professionals work in extremely taxing environments leading to recommendations of research networks to be formed.

## Conclusions

The data collected showed that the torture survivors in the five centres had high levels of exposure to torture events and high rates of clinical symptoms. Data can be collected by clinicians working in torture rehabilitation centres in low income countries, despite their very difficult working environments. However in order to provide effective care for torture survivors further research needs to evaluate fully the interventions used. To do this the clinicians need to overcome the methodological and logistical challenges described above and to be supported by research networks.

## Competing interests

The authors declare that they have no competing interests.

## Authors' contributions

MHO, BAR, MHA, AS, MF, NP, GS, CLS, FVP, LSC, ADH, EL, APAZ, LC, CLM, YAJ, ZA and HMC participated in the design of the study. MHO, BAR, MHA, AS, MF, NP, GS, CLS, FVP, LSC, ADH, EL, APAZ, LC, CLM, YAJ and ZA gathered the data for this study. CHS performed the statistical analysis of the data. HMC, CHS and SG participated in the coordination of the study and drafted the manuscript. All authors contributed to the outline of the manuscript. All authors read and approved the final manuscript.

## Authors' information

Authors of this paper are a mix of psychiatrists, psychologists, social workers and anthropologists. All except CHS, HMC and SG work directly with torture survivors on a day to day basis in one of the five torture rehabilitation centres that have participated in this study. CHS is a South Africa based research psychologist, with a special interest in the rehabilitation of torture survivors. HMC is a psychiatrist and SG is a social anthropologist both were working at the IRCT secretariat as a medic and the project manager respectively.
